# Explosion Pot. Chlor.

**Published:** 1874-11

**Authors:** 


*THE DRUGGISTS' CIRCULAR—August*, 1874:

Explosion of Chlorate of Potassa.—Mr. H. A. Wetzel, traveling agent, was engaged in pulverizing some chlorate of potassa, for the purpose of making some fire-works. While thus engaged the material exploded, bursting the mortar into a thousand pieces, sending the pestle up through the ceiling, and Mr. Wetzel was thrown backward, terribly injured. His right arm was badly burned and cut; the lower part of his abdomen and upper portion of his limbs were also badly cut and injured. But the most severe injury was to his eyes, which were so badly burned and hurt that it is feared he has lost his sight forever. A lad of some 12 years was standing by Mr. Wetzel's side, watching him, when the explosion took place, and was thrown back by the concussion and badly bruised, but not seriously. Another lad was standing about ten feet from Mr. Wetzel, and was blown against the partition. The ceiling of the room was badly shattered, and a large portion of the plastering fell to the floor. A short distance from where the mortar stood was a door with a glass window in it, leading to the office; this was badly shattered. Inside the office were Mr. Allaire and Mr. Woodward, engaged in writing. The explosion caused this room to be filled with dust, and knocked the pendulum off the clock. In the room immediately over the one where the explosion occurred was a lad sitting in a chair, and the concussion caused him to bound in the air. The cause of the explosion is not known, but a little sulphur, heat from friction, or even dust, would cause the material to explode.

